# Sexual dimorphism for juvenile body weight in chickens divergently selected for 8-week body weight

**DOI:** 10.3389/fphys.2024.1534334

**Published:** 2025-01-20

**Authors:** Paul B. Siegel, Christa F. Honaker

**Affiliations:** Virginia Tech, School of Animal Sciences, Blacksburg, VA, United States

**Keywords:** chicken, body weight, selection, dimorphism, homeostasis

## Abstract

There is a dearth of literature on the genetics of sexual dimorphism for juvenile body weight in meat-type chickens given its biological and economic relevance. Herein, we report the sexual dimorphism for 4- and 8-week body weights in White Plymouth Rock chicken lines that have undergone 67 generations of selection. Fluctuations in the degree of dimorphism occurred across generations, with greater dimorphism and less variation at 8 weeks than 4 weeks. Over the 67 generations, there was a significant decrease in the degree of dimorphism in the high line, with no changes in the low line. It is very difficult to genetically modify sexual dimorphism in a particular population using the currently available conventional tools, and nature possesses homeostatic mechanisms that maintain stasis in a population.

## Introduction

The weight of an organism at any point in its life can be considered a function of its genetic and environmental histories. Sexual dimorphism for body weight varies among gallinaceous birds, with male birds being heavier than female birds (e.g., chickens) or female birds being heavier than male birds (e.g., Japanese quail). In both cases, the degree of dimorphism at a specific age differs depending on the developmental and physiological aspects at the time of comparison. Furthermore, Rensch’s rule for sexual dimorphism for body weight in wild birds has been compromised in the case of the domestic chicken (Remeš and Székely, 2010).

Chickens were domesticated during the Neolithic period and have been amenable to artificial selection for various traits, including those focusing on sport, show, ceremony, and foods (Smith and Daniel, 1975). Chicken meat as food was primarily a result of traits selected for other purposes until the past century, when specialized breeding programs were designed for rapid juvenile growth (Siegel, 2014, 2023). This development was genetically facilitated by the availability of foundation stocks (Hanke et al., 1974), short generation intervals, and moderate heritability for juvenile body weights. Crosses between breeds and lines within the breeds allowed the breeders to protect their commercial products. The number of days to market weight has reduced dramatically (Havenstein et al., 2003; Zuidhof et al., 2014), and the age at market weight is currently measured in days instead of weeks. Because of sexual dimorphism in juvenile body weights, male and female birds attain their market weights at different ages; hence, sex-separate rearing is not uncommon and has become more popular. High-speed imaging and artificial intelligence tools have allowed autosexing of chicks at hatching through differences in the lengths of the primary and covert wing feathers, thereby replacing tedious human labor. Specific matings are needed, which are the result of sex-linked genetic effects (Chambers et al., 1994). Correlated responses, including increased feed intake (Carney et al., 2022), are associated with rapid juvenile growth and heavier body weights at younger ages. To address the potential cardiac, skeletal, and metabolic issues associated with excessive feed intake in the body weights of the breeders (adults), feeding programs to achieve the target body weights are readily available in management manuals provided by commercial distributors for their particular stocks.

Although the heritabilities for juvenile body weight in chickens can date back to several decades, as seen in the 176 summarized cases (Siegel, 1962), reports on the inheritance of sexual dimorphism *per se* are sparse. Sexual dimorphism for body weight in chickens can be viewed as the absolute or proportional differences between male and female birds. Thus, while the former may increase or decrease, the latter can remain constant or change. The inheritance of differences between the sexes for a common trait is confounded by multiple factors. Merritt (1966) and Mignon-Grasteau et al. (1998) provided valuable data and insights into the genetics involved as well as low heritability. Subsequently, Adedibu and Ayorinde (2011) reported the genetic differences in male to female body weight ratios at 4 and 8 weeks in one stock but only at 8 weeks in another stock. Maniatis et al. (2013) reported a genetic correlation of 0.91 for body weight between the sexes at 35 days, with heritabilities of 0.04 for the body weight differences and ratios. Although these results allow inference of little genetic variation for sexual dimorphism for juvenile body weights, differences among various breeds are well documented (American Poultry Association, 1947).

Starting from a common founder population of White Plymouth Rock (WPR) chickens, we developed two lines by divergent selection for a single trait, namely, body weight at 8 weeks (56 days) of age. After 67 generations of divergent selection, the two lines differed by more than 10-fold without any overlap in the body weight at selection age. Herein, we report the sexual dimorphisms for body weights at 4 and 8 weeks of age across the 67 selected generations as well as the founder population of these two lines.

## Materials and methods

### Animal use and care

All procedures and protocols used in this study as of 1997 have been approved by the Institutional Animal Care and Use Committee at Virginia Tech. Prior to 1997, the chickens were treated in a similar manner despite the university not having stated guidelines and protocols.

### Genetic lines

In 1957, a total of 15 male birds and 87 female birds from seven moderately inbred lines of early feathering WPR chickens were mated to produce a pedigreed population. This population provided the base for a selection experiment to produce two pedigreed lines, where one was selected for high body weight (HW) and the other for low body weight (LW) at 8 weeks post-hatching (Siegel, 1962; Dunnington and Siegel, 1996; Dunnington et al., 2013; Jambui et al., 2017). Although the selection of the parents to produce subsequent segregating generations was based on the body weights of the male and female chickens at 8 weeks of age, there were restrictions to avoid strict truncation. At hatching, caution was taken not to overrepresent progeny from a sire and from dams within a sire. Where possible, full- and half-sib matings were avoided when selecting parents for the subsequent generations. Over the 67 generations reported herein, adjustments were made to the breeding population. Within each line, the number of sires was increased from 8 to 12 at the fifth generation. Then, we increased these numbers to 14 sires and 56 dams in the twenty-sixth generation. Throughout the study, each sire was assigned four dams. The numbers of effective parents in these lines have been reported earlier (Marquez et al., 2010; Harrison et al., 2023).

### Husbandry

The incubation and grow-out facilities were the same across all generations. Thus, there is some redundancy here with prior works (Dunnington and Siegel, 1996; Jambui et al., 2017). One generation was produced each calendar year, with the hatch date being the first Tuesday in March. There was also a second “insurance” hatch 2 weeks later. In some generations, chickens from the second hatch were used to reproduce the next generation. On day 22 of incubation, the chicks were removed from the hatcher and wing-banded for pedigree. Starting from the seventeenth generation, the chicks were vaccinated for Marek’s disease after wing-banding. Brooding was conducted in the same building every year, and the only exception to this was in generations 54–63 where the LW chicks were brooded in battery cages until 2–4 weeks of age before being placed in the floor pens.

The lighting was continuous, with the litter being wood shavings on a concrete floor; the brooding was hot air supplemented with heat lamps. Water and an antibiotic-free mash diet containing a coccidiostat were allowed *ad libitum* throughout the study period. The diet was formulated to consist of 20% crude protein (CP) with a metabolizable energy (ME) of 2,685 kcal/kg. These values may have varied slightly from generation to generation owing to ingredient availability. Because of feed mill closing in the year 2022, we had to adjust the diet to 21% CP and 2,650 kcal/kg of ME.

### Traits measured and statistical analyses

In each generation, the individual body weights (g) were obtained at 4 and 8 weeks of age. Then, we calculated the means, standard deviations, and ratios (%) of female to male birds (lower the value, greater is the dimorphism) for each of the line-generation groups. Because the parental generation was a combination of several lines, it was analyzed separately from the two selected lines. Correlations between the means and generations were calculated for each line along with the regressions of means on the generations. For short-term associations, the generations were divided into six generational periods (2–12, 13–23, 24–34, 35–45, 46–56, and 57–67) and overall (1–67) for the correlation and regression analyses. The percentages were converted to arcsine of their square-root values for analysis. Although the subdivisions of the time trends indicate multiple uses of the same data (period within overall and overall), they provide short- and long-term insights.

## Results

**FIGURE 1:**
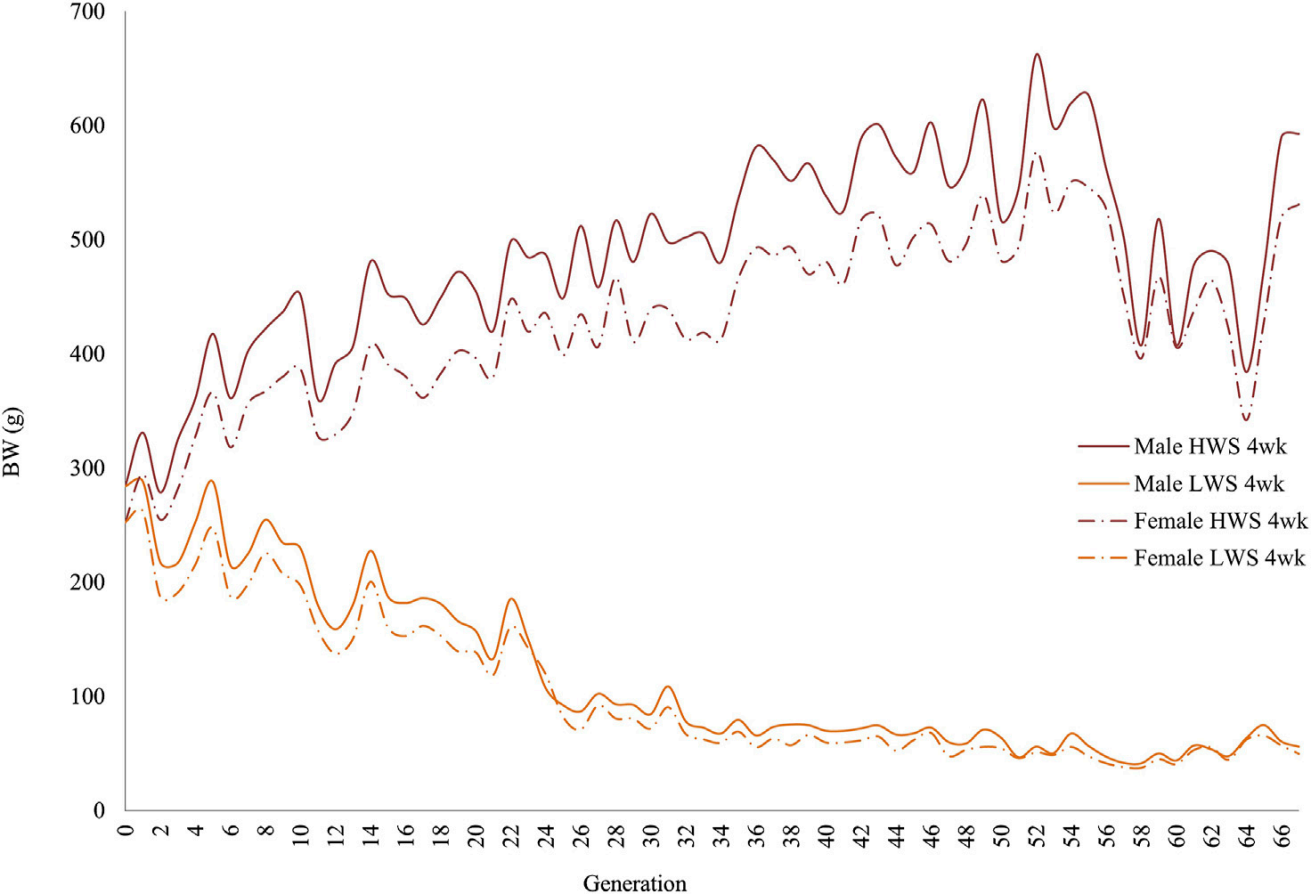
Generational mean body weights of the male and female chickens in the high (HWS) and low (LWS) body weight selected lines at 4 weeks of age.

**FIGURE 2:**
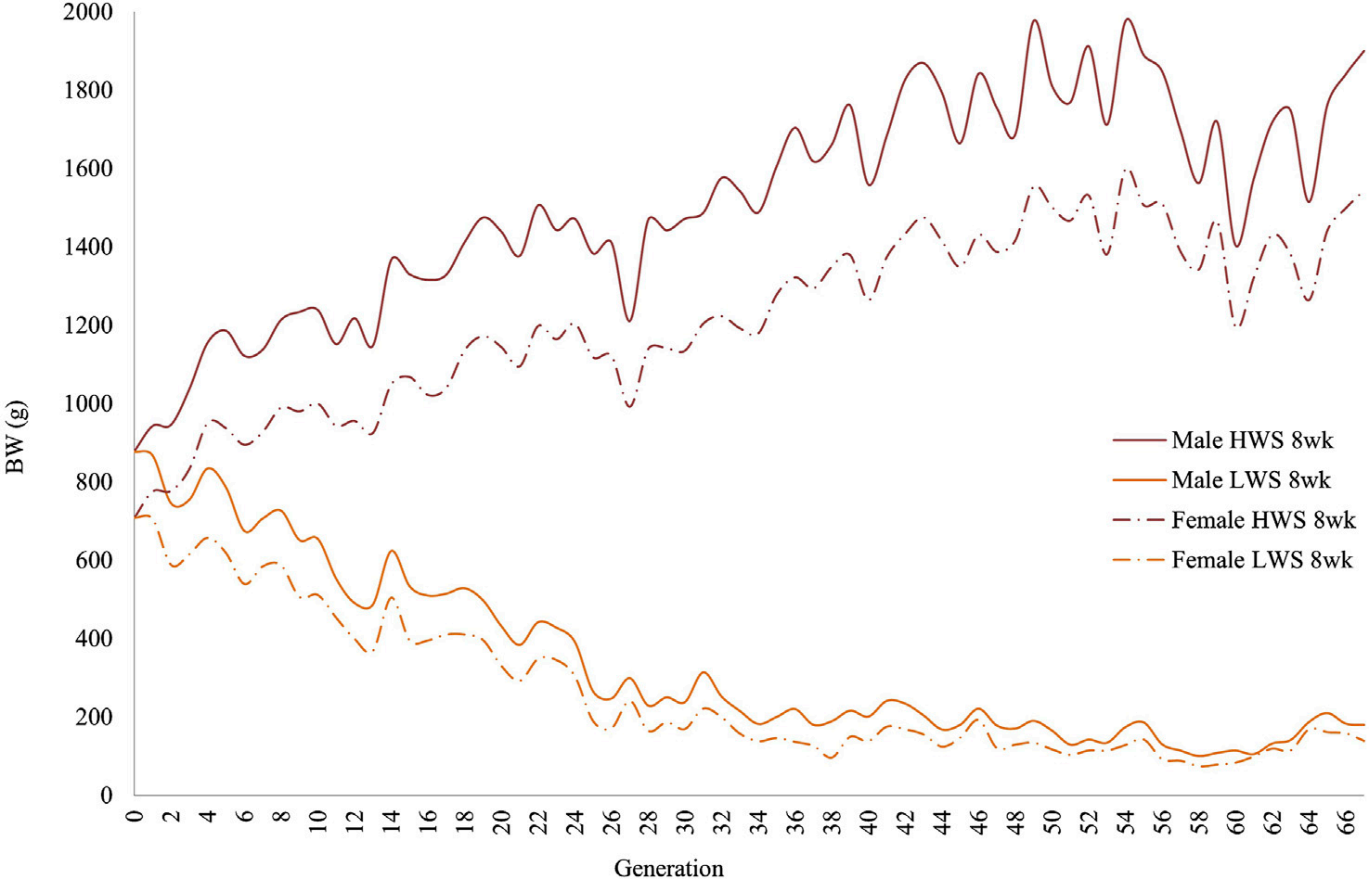
Generational mean body weights of the males and females in the high (HWS) and low (LWS) body weight selected lines at the 8-week selection age.

In both selected lines, the male chickens were consistently heavier than the female chickens at 4 and 8 weeks of age (Figures 1, 2). At 4 weeks, although the generational pattern was consistent for all generations of HW chickens, the degree of difference varied. For LW chickens, although the dimorphism followed the same pattern (males > females), some generations had slight differences between the male and female chickens. By 8 weeks, the magnitudes of dimorphism were more accentuated, with the male and female chickens consistently reflecting the within-generation environments.

**FIGURE 3:**
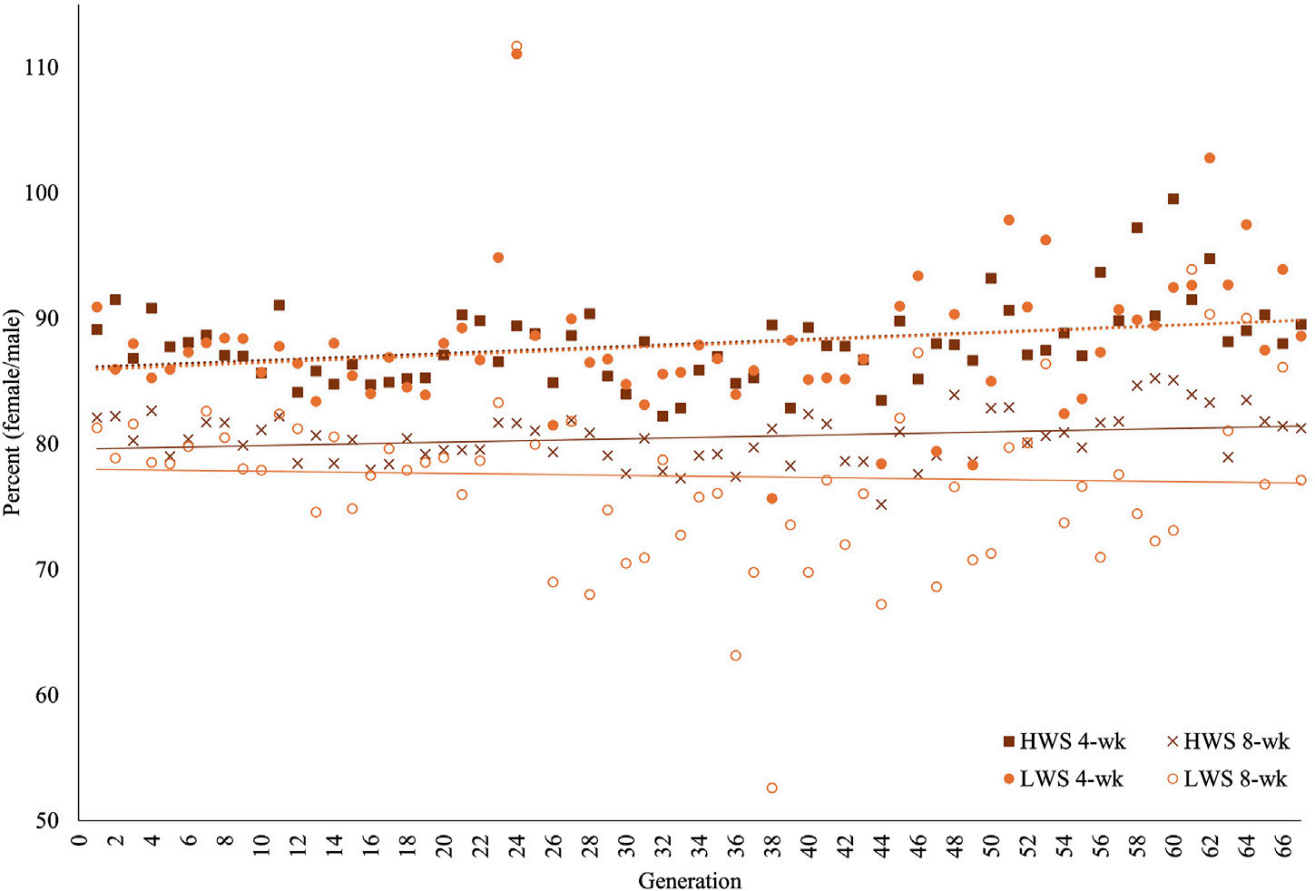
Percentage sexual dimorphisms ((female BW/male BW) × 100) for 67 generations of the high (HWS) and low (LWS) body weight selected lines at 4 and 8 weeks of age.

On a relative basis (with lower percentage indicating greater dimorphism), the greater dimorphism in the founder populations at 8 weeks (80.8%) than 4 weeks (88.9%) continued throughout the 67 generations in both HW and LW chickens (Table 1; Figure 3). When viewed across the 67 generations, there were generations when the relative sexual dimorphism for body weight appeared to increase or decrease. This “self-correction” pattern was observed at both ages, with the variation being greater in LW than HW chickens and more evident in the later than earlier generations (Table 1). The magnitude of variation in the HW chickens was consistently greater at 4 weeks than 8 weeks. In the LW chickens, the variation at 8 weeks was greater than that at 4 weeks, except for the F_13_–F_23_ and F_46_–F_56_ periods.

**TABLE 1:** Sexual dimorphisms (female BW/male BW expressed as a percentage ± SD^a^) by generational interval and overall at 4 and 8 weeks of age for the high (HW) and low (LW) body weight lines^b^.

Generation	4 weeks		8 weeks	
	HW	LW	HW	LW
F_2_–F_12_	88.04 ± 2.32	87.02 ± 1.18	80.88 ± 1.38	79.99 ± 1.76
F_13_–F_23_	86.44 ± 1.95	86.81 ± 3.30	79.61 ± 1.11	78.23 ± 2.53
F_24_–F_34_	86.42 ± 2.80	88.31 ± 7.92	79.66 ± 1.65	77.63 ± 12.17
F_35_–F_45_	86.75 ± 2.40	84.75 ± 4.29	79.38 ± 2.09	70.86 ± 7.97
F_46_–F_56_	88.70 ± 2.71	87.71 ± 6.59	80.73 ± 1.98	76.56 ± 6.30
F_57_–F_67_	91.63 ± 3.83	92.55 ± 4.39	82.81 ± 1.95	81.16 ± 7.66
F_1_–F_67_	88.01 ± 3.19	87.91 ± 5.42	80.53 ± 2.03	77.46 ± 7.71

^a^ Standard deviation (SD) is for each analyzed generational interval.

^b^ Parental (P_0_) generation dimorphisms were 88.9% and 80.8% at 4 and 8 weeks, respectively.

The same data were used in both the correlation and regression analyses because the evidence was unclear for cause–effect associations; the results obtained from these analyses were also similar. At 4 weeks, the regressions for both lines across all generations were essentially superimposed, while they were different at 8 weeks (Figure 3). Quantifications of these graphic patterns are provided in Tables 2, 3 for 4 and 8 weeks of age, respectively. At 4 weeks, there were generational periods wherein the dimorphism increased or decreased significantly only during the first half of this long-term selection experiment (Table 2). Although the generational interval differences were evident in both lines at 8 weeks, the patterns between them were different (Table 3). Overall, there was a significant reduction in dimorphism in the HW group but not in the LW chickens. Moreover, with the exception of the F_2_–F_12_ period, the standard errors of the regressions were one order of magnitude greater in the LW than HW chickens. These contrasting results between the two lines and ages are also seen in Figure 1.

**TABLE 2:** Correlations and regressions^a^ of the percentage sexual dimorphisms (female BW/male BW) by generational interval and overall at 4 weeks of age for the high (HW) and low (LW) body weight lines.

Generation	Correlation		Regression	
	HW	LW	HW	LW
F_2_–F_12_	−0.49	0.23	−0.35 ± 0.20	0.08 ± 0.12
F_13_–F_23_	0.63*	0.63*	0.37 ± 0.15*	0.62 ± 0.26*
F_24_–F_34_	−0.61*	−0.50	−0.52 ± 0.22*	−1.19 ± 0.69
F_35_–F_45_	0.18	0.12	0.13 ± 0.24	0.16 ± 0.43
F_46_–F_56_	0.41	0.02	0.34 ± 0.25	0.04 ± 0.66
F_57_–F_67_	−0.49	0.07	−0.57 ± 0.34	0.09 ± 0.44
F_1_–F_67_	0.34**	0.21	0.06 ± 0.02**	0.06 ± 0.03*

*Statistically significant at *p* ≤ 0.05.

**Statistically significant at *p* ≤ 0.01.

^a^ Standard error of regression is for each analyzed generational interval.

**TABLE 3:** Correlations and regressions^a^ of the percentage sexual dimorphisms (female BW/male BW) by generational interval and overall at 8 weeks of age for the high (HW) and low (LW) body weight lines.

Generation	Correlation		Regression	
	HW	LW	HW	LW
F_2_–F_12_	−0.27	0.22	−0.11 ± 0.13	0.12 ± 0.17
F_13_–F_23_	0.29	0.49	0.10 ± 0.11	0.37 ± 0.22
F_24_–F_34_	−0.71*	−0.49	−0.35 ± 0.12*	−1.80 ± 1.07
F_35_–F_45_	−0.07	0.41	−0.05 ± 0.21	0.98 ± 0.73
F_46_–F_56_	0.23	−0.09	0.14 ± 0.19	−0.16 ± 0.63
F_57_–F_67_	−0.53	0.31	−0.31 ± 0.17	0.72 ± 0.73
F_1_–F_67_	0.25*	−0.05	0.03 ± 0.01*	−0.02 ± 0.05

*Statistically significant at *p* ≤ 0.05.

^a^ Standard error of regression is for each analyzed generational interval.

## Discussion

The body weight of an organism at any point in time is a function of its genetic and nongenetic factors as well as past and present interactions. In multigenerational studies such as the one reported herein, epigenetic issues may also be relevant. The process across generations is dynamic, and social issues can be a factor when both male and female chickens are involved; however, these were precluded by our reproductive design. The chronological and physiological ages may differ, and these were documented early for the HW and LW lines (Katanbaf et al., 1988); prior to and since then, we have been aware of this dichotomy, as evidenced in the developmental stages for numerous traits (Washburn and Siegel, 1963; Siegel and Dunnington, 1985, 1987; Zelenka et al., 1988; Jambui et al., 2017). Thus, studying the inheritance of sexual dimorphism for body weight in chickens is a complex process.

As noted in the introduction, there is a vast amount of literature on the heritabilities for juvenile body weights for male and female chickens. Although these values are population specific, they may be similar (such as 0.28 for male and 0.29 for female chickens at 35 days) (Maniatis et al., 2013) or dissimilar (0.28 for male and 0.43 for female chickens at 8 weeks of age) (Mignon-Grasteau et al., 1998). The realized heritabilities at selection age for the first four generations of our experiment (male: female) were 0.23:0.21, 0.33:0.32, 0.35:0.27, and 0.31:0.28, respectively, with a small but consistent pattern and an unweighted mean of 0.30:0.27 (Siegel, 1962). These estimates are consistent with molecular analyses that showed that the 8-week body weights in these lines are influenced by many genes, each with small effects (Lillie et al., 2018). However, these findings do not address dimorphism *per se*, as such estimates involve two sets of individuals (males and females). The fact that the published heritability estimates of dimorphism *per se* of 0.08 (Mignon-Grasteau et al., 1998) and 0.04 (Maniatis et al., 2013) for juvenile body weights were low is not surprising, nor is the fact that the genetic correlation for body weight between the sexes was 0.91 (Maniatis et al., 2013). As noted by Merritt (1966), the magnitude of difference in heritabilities for male and female chickens is based on the contributions of the sire versus those of the dam per the sex chromosome, i.e., the sire is homogametic and dam is heterogametic. This means that the sex-linked genes are paired in male but not female progeny. Hence, the genetics of the sex chromosome is especially relevant when considering the inheritance of sexual dimorphism, particularly when comparisons involve populations selected in opposite directions from a common founder (such as the Virginia high and low weight lines). This is especially relevant when the genes at certain loci are at different frequencies and may be lost over time. Moreover, as the lines became more diverse over generations, the maternal and linkage effects may have become more relevant to the complexity of the differences in the 4- and 8-week body weights.

The Virginia lines are the WPR chickens, which is a meat-type breed (Hanke et al., 1974) first recognized by the American Standard of Perfection in 1888 (American Poultry Association, 1947). The WPR breed is a composite of the Cochin, Light Brahma, Black Minorca, Black Java, Langshan, and Dominique breeds (American Poultry Association, 1947; Guo et al., 2019). An analysis of the genealogy of the Virginia lines (Guo et al., 2019) showed that the overall proportions of autosomal contributions from the contributing breeds were similar. These contributions (HW:LW) were Cochins (30:32), Dominique (33:30), Black Java (26:27), Langshan (7:4), Light Brahma (4:7), and Black Minorca (0:0). However, when viewed at the individual chromosome level, there were major differences; this was evidenced by the contributions of the founder WPR breeds toward the sex chromosomes. For the W chromosome, the contributions came from just two of the founders, i.e., 49% Black Java and 51% Cochin, whose sexual dimorphism for juvenile body weights were 81% and 78%, respectively. In contrast, the percentage contributions to the Z chromosome (HW:LW) were from the Dominique (46:52), Black Java (28:21), Cochin (10:13), Light Brahma (11:9), Black Minorca (4:3), and Langshan (1:2) breeds, whose contributions to the dimorphism of juvenile body weights were 67%, 81%, 78%, 80%, 87%, and 81%, respectively (American Poultry Association, 1947). The changes in the contributions of these breeds toward the sex chromosomes occurred over many generations and were likely responsible for the cross-generational fluctuations that ultimately resulted in two and five of the original breeds contributing to the sex chromosomes.

Over the course of the 67 generations of selection for the high and low 8-week body weights, dynamic changes were noted to occur at the molecular and phenotypic levels for the males and females, with reductions in relative dimorphism at 4 and 8 weeks with selection for the HW line but not the LW line. Although the relative contributions from the founder WPR breeds were similar in both lines, there were large differences in the contributing stocks between the HW and LW lines in terms of the sex chromosomes. Across multiple generations, homeostatic mechanisms will often come into play, and the degree of dimorphism at a fixed age will be population-specific. It will be very difficult to genetically modify sexual dimorphism within a particular population using the conventional tools available at present. In breeding programs where the final product involves several closed populations, combining abilities may differ among the evaluated populations. The long-term divergent selection for 8-week body weight in the present study revealed the dynamic biological complexity of a trait that is ironically easy to measure.

## Data Availability

The raw data supporting the conclusions of this article will be made available by the authors without undue reservation.

## References

[B1] Adedibu I. I. Ayorinde K. L. (2011). Sexual dimorphism in predicting body weight of two broiler strains. Niger. J. Anim. Sci. 13 (1), 20–31.

[B2] American Poultry Association. (1947). The American Standard of Perfection. Davenport: American Poultry Association, Inc.

[B3] Carney V. L. Anthony N. B. Robinson F. E. Reimer B. L. Korver D. R. Zuidhof M. J. (2022). Evolution of maternal feed restriction practices over 60 years of selection for broiler productivity. Poult. Sci. 101 (10), 101957. 10.1016/j.psj.2022.101957 35973347 PMC9395665

[B4] Chambers J. R. Smith E. J. Dunnington E. A. Siegel P. B. (1994). Sex-linked feathering (*K, k+*) in chickens: a review. Poult. Sci. Rev. 5, 97–116.

[B5] Dunnington E. A. Honaker C. F. McGilliard M. Siegel P. B. (2013). Phenotypic responses of chickens to long-term, bidirectional selection for juvenile body weight -- historical perspective. Poult. Sci. 92 (7), 1724–1734. 10.3382/ps.2013-03069 23776258

[B6] Dunnington E. A. Siegel P. B. (1996). Long-term divergent selection for eight-week body weight in White Plymouth Rock chickens. Poult. Sci. 75, 1168–1179. 10.3382/ps.0751168 8893291

[B7] Guo Y. Lillie M. Zan Y. Beranger J. Martin A. Honaker C. F. (2019). A genomic inference of the White Plymouth Rock genealogy. Poult. Sci. 98 (11), 5272–5280. 10.3382/ps/pez411 31309227 PMC6863967

[B8] Hanke O. A. Skinner J. L. Florea J. W. (1974). American poultry history, 1823-1973, an anthology overview of 150 years. Madison: American Printing and Publishing, Inc, 1823–1973.

[B9] Harrison S. J. Siegel P. B. Honaker C. F. Lewis R. M. (2023). Population dynamics of a long-term selection experiment in White Plymouth Rock chickens selected for low or high body weight. Poult. Sci. 102 (5), 102575. 10.1016/j.psj.2023.102575 36907125 PMC10024231

[B10] Havenstein G. B. Ferket P. R. Qureshi M. A. (2003). Growth, livability, and feed conversion of 1957 versus 2001 broilers when fed representative 1957 and 2001 broiler diets. Poult. Sci. 82 (10), 1500–1508. 10.1093/ps/82.10.1500 14601725

[B11] Jambui M. Honaker C. F. Siegel P. B. (2017). Correlated responses to long-term divergent selection for 8-week body weight in female White Plymouth Rock chickens: sexual maturity. Poult. Sci. 96, 3844–3851. 10.3382/ps/pex224 29050442

[B12] Katanbaf M. N. Siegel P. B. Dunnington E. A. (1988). Organ growth of selected lines of chickens and their F_1_ crosses to a common body weight or age. Theor. Appl. Genet. 76 (4), 540–544. 10.1007/BF00260904 24232272

[B13] Lillie M. Sheng Z. Y. Honaker C. F. Andersson L. Siegel P. B. Carlborg Ö. (2018). Genomic signatures of 60 years of bidirectional selection for 8-week body weight in chickens. Poult. Sci. 97 (3), 781–790. 10.3382/ps/pex383 29272516

[B14] Maniatis G. Demiris N. Kranis A. Banos G. Kominakis A. (2013). Genetic analysis of sexual dimorphism of body weight in broilers. J. Appl. Genet. 54 (1), 61–70. 10.1007/s13353-012-0116-y 23001961

[B15] Marquez G. C. Siegel P. B. Lewis R. M. (2010). Genetic diversity and population structure in lines of chickens divergently selected for high and low 8-week body weight. Poult. Sci. 89, 2580–2588. 10.3382/ps.2010-01034 21076095

[B16] Merritt E. S. (1966). Estimates by sex of genetic parameters for body weight and skeletal dimensions in a random bred strain of meat type fowl. Poult. Sci. 45 (1), 118–125. 10.3382/ps.0450118 5911010

[B17] Mignon-Grasteau S. Beaumont C. Poivey J.-P. de Rochambeau H. (1998). Estimation of the genetic parameters of sexual dimorphism of body weight in ‘label’ chickens and Muscovy ducks. Genet. Sel. Evol. 30 (5), 481–491. 10.1186/1297-9686-30-5-481

[B18] Remeš V. Székely T. (2010). Domestic chickens defy Rensch's rule: sexual size dimorphism in chicken breeds. J. Evol. Biol. 23 (12), 2754–2759. 10.1111/j.1420-9101.2010.02126.x 21121089

[B19] Siegel P. B. (1962). Selection for body weight at eight weeks of age. Poult. Sci. 41 (3), 954–962. 10.3382/ps.0410954 4208544

[B20] Siegel P. B. (2014). Evolution of the modern broiler and feed efficiency. Annu. Rev. Anim.Biosci. 2, 375–385. 10.1146/annurev-animal-022513-114132 25384148

[B21] Siegel P. B. (2023). Broiler genetics and the future outlook. Front. Physiol. 14, 1150620. 10.3389/fphys.2023.1150620 36969607 PMC10031763

[B22] Siegel P. B. Dunnington E. A. (1985). “Reproductive complications associated with selection for broiler growth,” in Poultry breeding and genetics. Editors Hill W. G. Manson J. M. Hewitt D. (Harlow, Essex, UK: Longman Group Ltd.), 59–72.

[B23] Siegel P. B. Dunnington E. A. (1987). Selection for growth in chickens. Crit. Rev. Poult.Biol 1, 1–24.

[B24] Smith P. Daniel C. (1975). The chicken book. Boston: Little and Brown Co.

[B25] Washburn K. W. Siegel P. B. (1963). Influence of thiouracil on chickens selected for high and low body weights. Poult. Sci. 42, 161–169. 10.3382/ps.0420161

[B26] Zelenka D. J. Dunnington E. A. Cherry J. A. Siegel P. B. (1988). Anorexia and sexual maturity in female white rock chickens. I. Increasing the feed intake maturity in female white rock chickens. 1. Increasing the feed intake. Behav. Genet. 18 (3), 383–387. 10.1007/BF01260938 3219115

[B27] Zuidhof M. J. Carney V. L. Korver D. R. Robinson F. E. (2014). Growth, efficiency, and yield of commercial broilers from 1957, 1978, and 2005. Poult. Sci. 93 (12), 2970–2982. 10.3382/ps.2014-04291 25260522 PMC4988556

